# Supplementary material for: Alcohol unleashes homo economicus by inhibiting cooperation
Source: PLoS One. 2021 Jun 22;16(6):e0253296. doi: 10.1371/journal.pone.0253296 (PMC8219160; doi:10.1371/journal.pone.0253296)
Supplement: Attachment — Submitted filename: Reply to referees 4-1-21 short.docx [file pone.0253296.s001.docx]

My coauthors and I thank the editor and the two reviewers for very useful comments. We have addressed each one in this letter and have revised the ms to reflect your comments. We are grateful for your efforts that have improved our paper.

Reply to Editor:

*1. Regarding power calculations I am not asking for new experimental sessions. It would be enough if you just recognize that certain results might be taken cautiously due to the lack of power (since you have many treatment and quite limited sample).*

We have added a power of test calculation and modified the Discussion to note some tests may be low powered.

****************

Reviewer #1: The paper is well done and the conclusions are interesting. I have only two small comments.

*1. One is that I do not know why the authors did the two treatments where participants drank alone or in a group. This ends up not making a difference, but I suppose they conjectured it could have been different and I would like to understand this.*

The revised ms. now clarifies that the experimental design sought to isolate the effects of alcohol from the effects of socializing. The isolation effect only impacted the physiologic stress response as noted in the Results.

*2. The other point is that the closest study I can see is the one of of Corazzini, Filippin & Vanin, 2015. I am surprised that they chraracterize it as "Alcohol use has been reported to weakly reduce charitable donations." I was curious and downloaded the paper. It seems like the donations to the both the charity MSF and the journal are reduced by about a half of the baseline. So in a sense it is very much in line with what the authors find here, if anything a bit higher. One added value of this study in my view is to do it in a strategic setting, on the one hand. Did the authors think the strategic setting would change something? If so, why? If not, what have we learnt? The other added value is to check the mediation of stress, which the other study did not check. I have a sense this is the major contribution, and perhaps the authors could highlight it a bit more.*

We thank this reviewer for very useful comments. The Corazzini, Filippin & Vanin, 2015 paper is the most similar to our and is the reason we sent our paper to PLoS One. CFV state that " Column (1) confirms that donations are higher in the NO-ALC group, but the difference is not statistically significant." If those authors slice the data thin enough, they get an effect of MSF, but not donations overall. This is the basis for our statement. We appreciate your comments on the strategic setting and our finding for stress. We have added additional text to the revised ms. on both these points.

***********

Reviewer #2:

*1. The sample is clearly too small. 128 individuals, split into 2 treatements (control and intervention), plus in 2 again (drink alone or drink with 3 people). I would expect more sessions to be conducted.*

A power of test calculation is now added to the revised ms showing the majority of statistical tests have high power. Most of the analysis uses the full sample to keep the power of tests high, identifying subconditions such as drinking alone with binary indicator variables. With the current pandemic and on advice from the editor, we are unable to conduct additional sessions of this experiment. We have added a cautionary note in the Discussion that segmenting the sample will reduce the power of the statistical tests.

*2. I would need more details about the sessions. It is said that a session consists of 12 people, how were exactly composed the sessions since with 128 people you cannot have only sessions of 12?*

The paper states that "Typical sessions included 12 participants." Some sessions were smaller or larger due to dropouts and a research assistant filled in the group of 4 with the fill-in data not analyzed.

*3. Why were there mixed groups? There is a large litterature showing that men and women react differently to alcohol and behavioural tasks, why not making groups of females only and of men only?*

The revised ms now clarifies that we sought to capture what typically happens when people go to a bar.

*4. It is said there was no deception, however some participants had placebo and it is said they were pushed to believe they will have alcohol. So I am sorry, but I cannot understand how this was not deception?*

Participants were informed they would consume alcohol or placebo, hence no deception.

*5. It is not clear if participants knew the task in advance, while drinking, and if they knew there will be a task at all?*

The revised ms. clarifies that during the consumption phase, participants did not know what tasks they would do later.

*6. It is explained that alcohol makes people act on impulse, but economic papers show that when acting on impulse, people are more cooperative. So I would appreciate a discussion.*

The revised ms. Discussion now brings in more literature on impulsivity and economic decisions. Thank you for this suggestion.

*7. It is said that alcohol motivates the desire for immediate reward, but this reward could be warm glow or something like this, so, again, I would need a discussion.*

We have added a cite to the Discussion on this.

*8. There is a huge number of papers explaing that pre-play interaction has positive effects on cooperation, here the pre-play has not, I need a discussion.*

Great suggestion, we have added this to the Discussion. The role of pre-play was reduced by randomly matching participants in groups of four for the PGG.

*9. It is said that when moving from drinking in groups of 4 to playing the public good game they did not stay in the same group. Why ?*

Revised ms. clarifies in Methods that the PGG used random matching of participants --the goal was to reduce the effect of pre-play interactions.

*10. I do not see any results about the differences between those who had drinks alone and in a group.*

We now mention this nonfinding on PPGg and PPGc to the first paragraph of the Results.

11. It is sais they made 4 decisions, that means there were 4 rounds in the public good game?

Correct, with random rematching.

*12. I see the experiment as very invasive with all the measures etc...so this would compensate on the social effect of drinking, right? The situation is not "normal" and this is not how cooperation decisions are taken "in real life" after a drink. So this can explain the results as participants understand that they are expected to be inhebriated and their actions under this condition will be studied. So this can induce behavior. I would need some discussion.*

The study did not claim to replicate real life. Rather, we sought to establish the role of a commonly used drug (alcohol) on cooperation. My lab has published many studies using blood draws to establish neurochemical mechanisms affecting behavior and choice data are very similar to that found without blood draws. We now mention this in the Discussion with a citation.
