# Supplementary material for: Alcohol unleashes homo economicus by inhibiting cooperation
Source: PLoS One. 2021 Jun 22;16(6):e0253296. doi: 10.1371/journal.pone.0253296 (PMC8219160; doi:10.1371/journal.pone.0253296)
Supplement: Attachment — Submitted filename: Reply2 to R2 5-29-21.docx [file pone.0253296.s002.docx]

We thank R2 for taking the time to offer additional suggestions to improve our ms. We looked at each one in detail and have revised the ms accordingly. My coauthors and I believe this revision addresses the suggestions you have made and herein we detail the changes. We expect you will find this revision satisfies your remaining questions.

*Power*. As we note in the cover letter, the bulk of our analyses use the full N=124 data set with indicators for treatment and socializing so these analyses are not underpowered. In addition, as suggested by the editor, we have done a panel data analysis with the full set of n=496 behavioral observations (rather than averaging PGG behavior). The revised ms. now reports the panel data analysis showing that alcohol continues to reduce PGG contributions. The editor also recommended analyzing alcohol's effects on round 1 and round 4 contributions. Again, the results for alcohol reducing PGG contributions are sustained as the paper now reports. We appreciate these excellent suggestions that show the robustness of the findings.

For these reasons, we disagree that more data is needed. The only underpowered tests reported are for free riders and the effect of drinking alone, both secondary analyses that the paper includes but are not core findings. Further, both these findings are statistically significant even with lower power. In addition, the drinking alone condition was only significant in affecting the stress response and was not otherwise used in the analysis so that sample is not split in half due to this. Indeed, we believe the hypothesis of whether it is alcohol or socializing that affects behavior was worth testing and reporting even if it did not affect behavior.

*Replacements*. There were only 4 instances of RAs filling in and these data were not analyzed as the paper states. The Methods of the revised ms now expands on this and clarifies that RAs did not drink and could not be identified by participants as decisions were anonymous with the RA filling in a PGG group entering data from the control room. Since participants never received feedback on others' contributions, the revised paper emphasizes there is no way for this filling to affect participants' decisions.

*Mixed sexes and design approach*. This was a design choice to capture the most common effects for an already complicated experiment (e.g. we hired a phlebotomist to do 248 blood draws, then we processed these tubes to isolate plasma that was stored in an ultracold freezer until sent for analysis, mixed drinks, measured BAC multiple times, etc.). My lab is focused on identifying mechanisms producing behavior which is why we took blood samples before and after drinking. Lab studies balance what people do outside the lab (e.g. drink) with the precision that lab studies offer in measuring behavior and the mechanisms that produce them. There are many ways to study the effects of drinking on cooperation, but our finding of stress and negative affect are unique to the literature and help explain why alcohol reduces cooperation. We have added to the Discussion that our result may not generalize to single sex groups or older individuals as you noted. Our hope is that this study provokes others to explore different ways to study this important topic.

*Deception*: The revised Methods now clarifies that the instructions to participants stated that only one-half would drink alcohol while the others would receive a placebo. The masking of the placebo followed a protocol in the cited alcohol literature as we now emphasize.

*Tasks*. The revised ms. clarifies that participants only knew they would "make decisions," but not what decisions they would make.
